# Supplementary material for: Diagnostic Accuracy of Insulinoma-Associated Protein 1 in Pulmonary Neuroendocrine Carcinomas: A Systematic Review and Meta-Analysis
Source: Cancers (Basel). 2025 Jul 31;17(15):2544. doi: 10.3390/cancers17152544 (PMC12345728; doi:10.3390/cancers17152544)
Supplement: Supplementary file 1 [file cancers-17-02544-s001.zip › Table S2. Search strategy for systematic review.pdf]

Diagnostic accuracy of Insulinoma-associated protein 1

in pulmonary neuroendocrine carcinomas:

a systematic review and meta-analysis

Risa Waki, Saya Haketa, Riona Aburaki, Nobuyuki Horita

<Supplementary File>

Table S2. Search strategy for systematic review.

Data base	Search formula	Number of records identified
PubMed	(insulinoma-associated protein 1 OR INSM1 OR INSM-1) AND (lung OR pulmonary OR respiratory OR thoracic OR bronchial OR bronchogenic OR tracheal OR alveolar)	N=111
WOS	TS=(insulinoma-associated protein 1 OR INSM1 OR INSM-1) AND TS=(lung OR pulmonary OR respiratory OR thoracic OR bronchial OR bronchogenic OR tracheal OR alveolar)	N=151
EMBASE	(insulinoma-associated protein 1 OR INSM1 OR INSM-1) AND (lung OR pulmonary OR respiratory OR thoracic OR bronchial OR bronchogenic OR tracheal OR alveolar)	N=256
