# Supplementary figures and images for: Diagnostic Accuracy of Insulinoma-Associated Protein 1 in Pulmonary Neuroendocrine Carcinomas: A Systematic Review and Meta-Analysis
Source: Cancers (Basel). 2025 Jul 31;17(15):2544. doi: 10.3390/cancers17152544 (PMC12345728; doi:10.3390/cancers17152544)

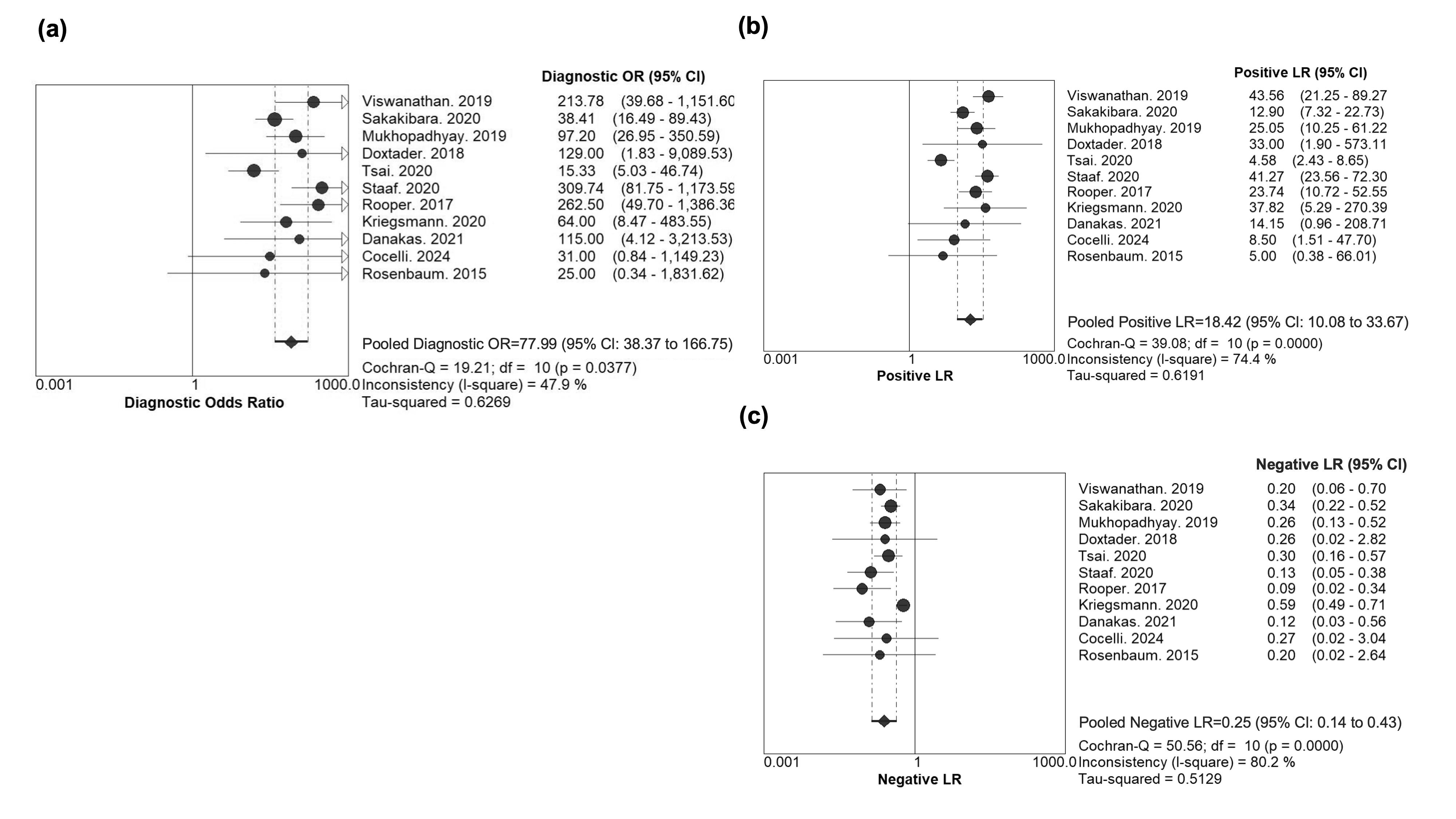

Supplement: Supplementary file 1 [file cancers-17-02544-s001.zip › Figure S1. DOR, PLR, and NLR in NSCLC model.jpg]

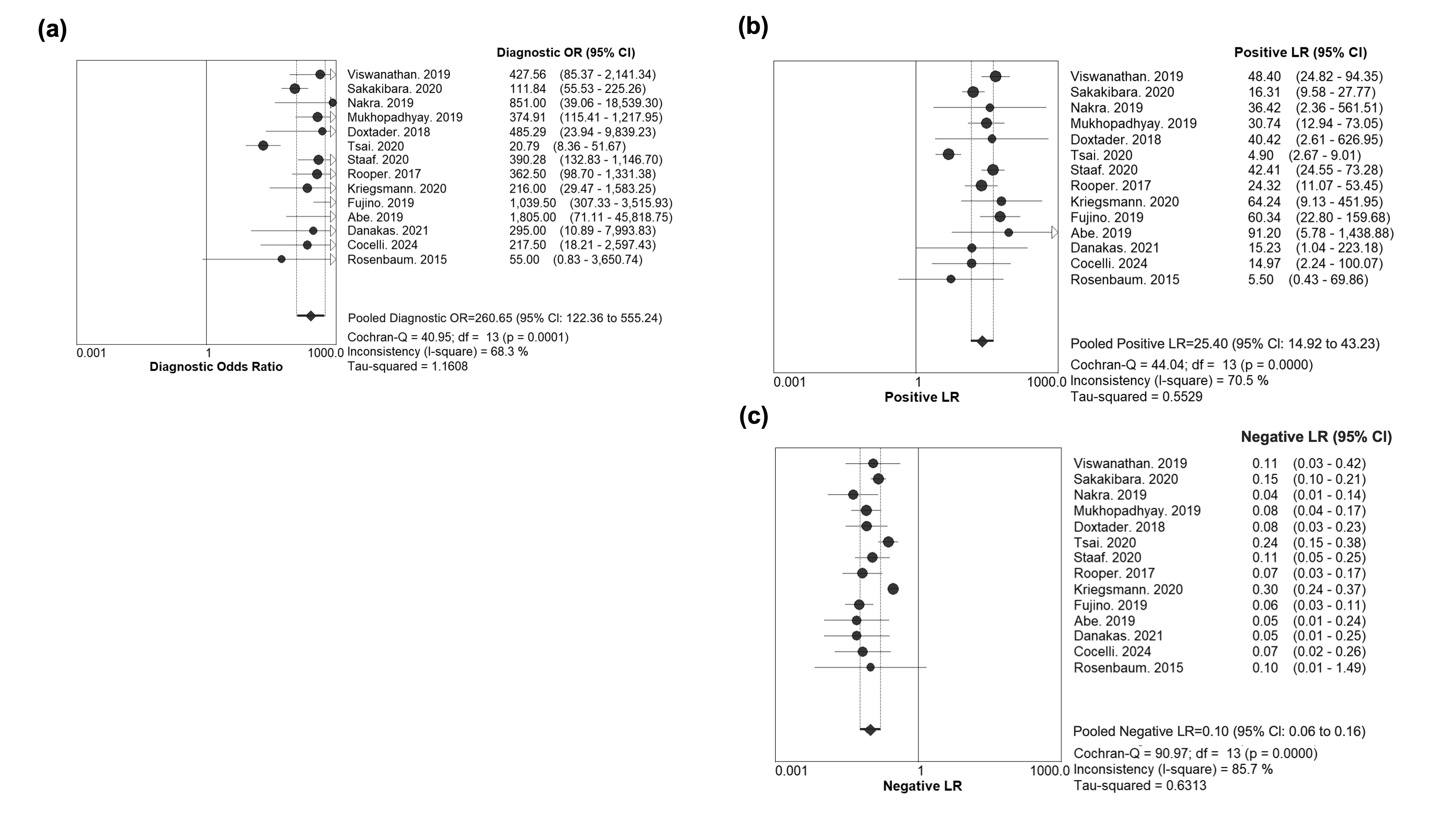

Supplement: Supplementary file 1 [file cancers-17-02544-s001.zip › Figure S2. DOR, PLR, and NLR in lung cancer model.jpg]
